# Adaptive and innate immune pathogenesis of bullous pemphigoid: A review

**DOI:** 10.3389/fimmu.2023.1144429

**Published:** 2023-03-10

**Authors:** Tianmeng Yan, Zhenying Zhang

**Affiliations:** ^1^ Department of Dermatology, The University of Hong Kong Shenzhen Hospital, Shenzhen, China; ^2^ Department of Dermatology, The Eighth Affiliated Hospital of Sun Yat-sen University, Shenzhen, China

**Keywords:** bullous pemphigoid, adaptive immunity, innate immunity, crosstalk, pathogenesis

## Abstract

Bullous pemphigoid (BP) is an autoimmune blistering disease that primarily affects elderly individuals. The presentation of BP is heterogeneous, typically manifesting as microscopic subepidermal separation with a mixed inflammatory infiltrate. The mechanism of pemphigoid development is unclear. B cells play a major role in pathogenic autoantibody production, and T cells, type II inflammatory cytokines, eosinophils, mast cells, neutrophils, and keratinocytes are also implicated in the pathogenesis of BP. Here, we review the roles of and crosstalk between innate and adaptive immune cells in BP.

## 1 Introduction

Bullous pemphigoid (BP) is an autoimmune blistering disease that primarily affects the elderly. As a result of aging of the population, the incidence of BP has increased (1, 2) to 2.5–42.8 cases/million/year (3). The presentation of BP is heterogeneous, typically manifesting as lesions such as tense blisters and erythematous and urticarial plaques. Subepidermal separation with inflammatory infiltrates comprising eosinophils, neutrophils, and lymphocytes has been observed (4). Linear deposition of autoantibodies and/or complement 3 (C3) along the dermo-epidermal junction has been detected by immunofluorescence analysis (4).

The pathogenesis of pemphigoid is unclear, but autoantibodies to the hemidesmosome are implicated. Subepidermal blister formation with obvious inflammatory-cell infiltration is a hallmark of BP but not pemphigus disease (5). The pathogenesis of BP involves various immune cells and factors, including B cells (6), T cells (7), complement cells (5), mast cells (8), neutrophils (9), and eosinophils (10). The pathogenetic role of interactions among the aforementioned types of inflammatory cells is unclear. Here, we review the immune cells and cytokines implicated in the pathogenesis of BP.

## 2 Immune cells

### 2.1 Adaptive immunity

#### 2.1.1 B cells and autoantibodies

B cells are thought to play a critical role in the pathogenesis of BP, which is confirmed by the efficiency of B-cell depletion therapy for refractory pemphigoid (11). It can also be supported by the increased expression of BAFF (B-cell activating factor) in BP (12, 13) and that lower peak serum BAFF levels after rituximab treatment in BP patients predict relapse and a need for earlier intervention (12, 13). Most BP patients have serum autoantibodies to the BMZ, which are termed BP180/type XVII collagen/BPAG2 and BP230/BPAG1; these are key components of hemidesmosomes, which mediate adhesion of the epidermis to the dermis. The pathogenic autoantibodies implicated in BP are produced by B cells. The mechanism by which autoreactive B cells are activated to produce autoantibodies has been extensively investigated.

BP180 is a transmembrane protein of the hemidesmosomes in basal keratinocytes. The extracellular domain of BP180 contains 15 interrupted repeated collagenous domains, and its structure consists of a globular head, central rod, and flexible tail. BP180 is inserted into the lamina densa *via* the rod domain and loops back through the lamina densa *via* its N-terminal tail (14). Several epitopes of BP180 have been identified, and differential epitope recognition is associated with clinical severity (15). The extracellular portion of the 16th non-collagenous domain (NC16A) of BP180 is the main epitope targeted by autoantibodies. IgG autoantibodies deplete BP180 in cultured normal human keratinocytes, thereby reducing their adhesion (16). The injection of mice with rabbit anti-mouse BP180 antibody induces blisters (17). Anti-human BP180 IgG produced by immunized mCol17^+/-^ mother mice can induce BP lesions in their neonates whose skin expressed human but not mouse COL17 (18, 19). BP180 NC-16A-specific IgG autoantibodies are of the IgG1 and IgG4 subclasses (20). After binding to BP180-NC16A antigen, IgG1 recruits C3 to activate the complement cascade (21). In contrast, anti-NC16A IgG4 autoantibodies are complement-independent (22). IgG4-antigen complexes recruit various inflammatory cells, which release cytokines that induce the separation of the BMZ and local inflammation. IgG4 autoantibodies may block IgG1 and IgG3 by binding to NC16A, thereby inhibiting inflammation (23). IgG1 and IgG4 autoantibody titers are implicated in disease activity in BP (20). BP230, a cytoplasmic protein of the hemidesmosomes, is a plakin-family protein consisting of N-terminal, C-tail, globular, and central rod domains. BP230 serves as a bridge by binding to BP180 *via* its N-terminal domain and to the intermediate filament-binding domain *via* its C-tail domain. Anti-BP230 IgG autoantibodies, which are of the IgG1 and IgG4 subclasses, are present in most BP patients and typically target the C-tail and intermediate filament domains (24). The pathogenic role of anti-BP230 autoantibodies is unclear. The anti-BP230 IgG titer is not associated with disease severity but is implicated in atypical BP phenotypes (25, 26). In an anti-BP230 mouse model, anti-BP230 autoantibodies induced blister formation in the absence of BP180 (27).

IgE-mediated autoimmunity may be involved in BP blister development (28, 29). IgE autoantibodies target the intracellular domain of BP180. IgE autoantibodies induce BP180 internalization from the surface of basal keratinocytes, thereby suppressing their adhesion (30). IgE deposition along the dermo-epidermal junction was detected in perilesional skin (31–33). Circulating total IgE is elevated in 60%–85% of BP patients. BP180 IgE was detected in 22%–100% of BP patients (34, 35). BP230-specific IgE is prevalent in BP (35, 36). The high IgE autoantibody level in BP patients may necessitate aggressive treatment (37). However, the relationship between BP180NC16 IgE and disease severity is unclear (32, 38).

IgE production and its downstream effects are regulated by a complex network of cell-bound and soluble receptors, such as FcϵRI and CD23/FcϵRII. The expression of CD23 and FcϵRI on circulating eosinophils, mast cells, basophils, and B cells is increased in BP patients (39–42). Similarly, soluble CD23 expression is elevated in serum and blister fluid from BP patients (43, 44).

#### 2.1.2 T cells

Autoreactive T cells have been detected in the peripheral blood of BP patients with active disease but not in the blood of those in remission (45, 46). T-cell activation by an autoantibody molecule can induce a variety of responses of B cells to a cross-reactive version of the original epitope (47). In BP, CXCL12, which is derived from infiltrated CD3^+^ T cells in lesions, induced the chemotaxis and accumulation of CXCR4^+^ B cells by activating the transcription factor c-Myc, thus promoting B-cell differentiation into autoantibody-secreting cells and facilitating autoantibody production (48). CD3^+^ T cells are categorized as cytotoxic (CD8^+^) or helper (CD4^+^) T cells (Th cells). CD4^+^ Th cells play a central role in activating immune cells in BP. CD4^+^ T cells are classified as Th1, Th2, Th17, T follicular helper (Tfh) cells, or regulatory T (Treg) cells depending on the inflammatory reaction (49). The Th1/Th2, Th17/Treg, and Tfh/Treg ratios are important for immune tolerance (50–53).

Th2 cells and IL-4 play a role in the pathogenesis of BP by promoting autoantibody production by B cells (54). B-cell activation by Th2 cells or surface-clustered immunoglobulins bound to the epitope of the antigen initiates this process (55). P2 (492–506 aa, VRKLKARVDELERIR) and P5 (501–515 aa, ELERIRRSILPYGDS), which are both peptides of BP180 NC16A (the main antigen in BP), are important for IL-4 production by Th2 cells and autoantibody production by B cells (54). The activation of Th2 cells in BP is consistent with predominant IgG4 autoantibody production: IL-4 regulates IgG isotype switching, thereby amplifying IgG4 production (56). IL-4 also promotes IgE isotype switching to stimulate IgE production (57, 58). IL-4 and IL-13 are mainly secreted by Th2 cells. The efficacy of dupilumab (autoantibody against IL-4 and IL-13R) in BP implicates type II inflammation in its pathogenesis (59). Moreover, autoreactive Th2 and Th1 cells regulate the autoantibody response to the immunodominant sequences of BP230 (46). In experimental BP models, CD4^+^ T cells were crucial to promoting the production of pathogenic anti-hCOL17NC16A IgG, leading to active disease (60).

Treg cells maintain peripheral immune tolerance by suppressing autoreactive T cells (61). The contribution of Treg cells to BP is controversial. In a mouse model, Tregs alleviate pemphigoid lesions by altering the migratory capabilities of myeloid cells (62), and an absence of Treg cells leads to pemphigoid lesions (63). In another mouse model, Treg cells suppressed steady-state autoimmune reactions to BP230 and COL17 (64). CD4^+^ CD25brightFOXP3^+^ Treg-cell expression is increased in peripheral blood and skin lesions from BP patients (65, 66). In conventional BP patients, the expression levels of total Tregs and Treg subsets were increased before, and decreased after, systemic corticosteroid treatment. The expression of CD45RA^−^Foxp3^hi^ effector Treg cells is positively correlated with disease severity in conventional BP, and CD45RA^+^Foxp3^lo^-naive Treg cell expression is positively correlated with disease severity in DPP-4i related BP (67). Differences in results among studies may be explained by the use of different markers of Tregs.

Tfh cells promote the production of high-affinity autoantibodies by B cells in germinal centers. CXCR5, ICOS, Bcl-6, CD40 ligand (CD40L), and PD-1 are membrane-bound markers of Tfh cells (68). IL-21 is preferentially expressed by Tfh cells and regulates humoral responses by modulating B-cell proliferation and class switching (69). BP patients have high plasma levels of Tfh cells and IL-21 and increased CXCR5 expression in lesions (70). In addition, CXCL13, which recruits CXCR5^+^ Tfh cells, is increased in BP lesions and peripheral blood and is positively correlated with the serum anti-BP180-NC161 titer (71). The inhibition of Tfh-cell factors (e.g., CD40L, PD-1, ICOS, and IL-21) suppresses autoantibody production (72–75).

The role of Th17 cells in the pathogenesis of BP is controversial. Th17 cells promote autoimmune pathology by secreting IL-17, IL-21, IL-22, IFN-γ, and granulocyte-macrophage colony-stimulating factor (GM-CSF) (76). Two single-nucleotide polymorphisms, rs2201841 and rs7530511, of *IL*‐*23R* encoding the receptor for IL-23, which is an upstream cytokine of IL-17, are associated with BP (77). IL-17A^+^CD4^+^ lymphocytes were elevated in BP peripheral blood and skin lesions (53, 78). The absence of the NC14A domain of BP180 in mice induced an IL-17-associated autoimmune response against the cutaneous basement membrane, which was ameliorated after anti-17A treatment (79). IL-17A-deficient mice were protected against autoantibody-induced BP (78). IL-17 upregulates CXCL10, which increases matrix metalloproteinase-9 (MMP-9) secretion in monocytes and neutrophils, and promotes blister formation (80, 81). Clinical trials with biologics targeting the IL-17/IL-23 axis (NCT04117932 and NCT04465292) were conducted in BP patients.

### 2.2 Innate immunity

#### 2.2.1 Eosinophils

Eosinophilic infiltrates and peripheral eosinophilia are features of BP and are associated with disease severity and outcome (82, 83). Eosinophil degranulation is prominent in early BP lesions and is essential for blister formation (84). The localization of eosinophils to the BMZ is dependent on IgG and complement fixation (85). However, the interaction of eosinophils with IgE may induce their degranulation (86). Eosinophils highly express FcϵRI, which promotes their interaction with BP IgE autoantibodies (which results in eosinophil degranulation and blister formation) (28, 40). Eosinophils also promote initiation of the coagulation cascade (87). In BP patients treated with omalizumab, an autoantibody targeting IgE, disease severity was closely related to peripheral eosinophils, but not IgG (29).

Eosinophils exposed to eotaxin, GM-CSF, IL-5, IFN-γ, and thymic stromal lymphopoietin cytokine (TSLP) promote the release of toxic granule proteins, including major basic protein (MBP), eosinophil cationic protein (ECP), eosinophil peroxidase (EPO), and MMP-9; in turn, these induce a local inflammatory response (84, 88–91). Eosinophil extracellular traps (EETs), which have a web-like structure containing nuclear DNA and proteins, were also discovered in BP lesions (92). IL-5, which is elevated in blister fluid, is essential for toxic protein release by eosinophils and the separation of keratinocytes (93). ECP, MBP, and EPO are increased in BP lesions and plasma and directly promote the separation of keratinocytes (94). In addition, ECP and eosinophil-derived neurotoxin (EDN) are decreased in plasma after immunosuppressive treatment, suggesting that these markers are associated with disease activity (95). An initially low level of ECP may promote remission within the first year (95). Benralizumab, a humanized IgG1κ monoclonal autoantibody against the IL-5R α subunit, and bertilimumab, a humanized monoclonal autoantibody targeting eotaxin-1 (CCL-11), are currently being evaluated in clinical trials as treatments for BP (NCT02226146 and 04612790).

#### 2.2.2 Neutrophils

Neutrophils infiltrate BP skin lesions and release proteolytic enzymes and reactive intermediates to promote inflammation. A BP model suggests that neutrophils are a determinant of disease phenotype (96, 97). The cytokines and proteases secreted by neutrophils include myeloperoxidase (MPO), neutrophil elastase (NE), MMP-9, and neutrophil-derived nicotinamide adenine dinucleotide phosphate (NADPH). These cytokines degrade the extracellular matrix and split dermal–epidermal junctions, thus exerting an immunomodulatory effect in autoimmune diseases (81, 98, 99). The formation of neutrophil extracellular traps (NETs), like EETs, is increased in BP peripheral blood and lesions and correlates with disease activity (100, 101). *In vitro*, BP180-NC16A antigen-antibody complexes can induce NETosis, releasing NETs through a cell death process (9). Elevated NETs in BP patients boost autoantibody production by inducing B-cell differentiation into plasma cells, an effect mediated by MAPK P38 cascade activation (9).

#### 2.2.3 Mast cells

Mast cells accumulate and degranulate in early BP lesions (8). The role of mast cells in pemphigoid is debated (102, 103). FcϵRI, an IgE receptor expressed on mast cells, may induce IgE-mediated inflammation, urticarial plaques, skin edema, and eosinophil accumulation and activation (8). Mast cells with IgE and BP180 peptides are present in BP lesions and induce mast cell degranulation (104). Multiple inflammatory cytokines and proteases are released from mast cell granules following their activation. Tryptase, a marker of mast cells, is increased in blisters of BP and related to the BP autoantibody titer to the BMZ. Moreover, the plasma level of tryptase is related to BP autoantibodies (105). IL-5, released by mast cells, promotes eosinophil accumulation and activation in BP. In BP models, activated mast cells release mouse mast cell protease-4 (MCP-4), a homolog of human chymase, which activates MMP-9 and cleaves BP180 (106). MCP-4 also activates NET release by neutrophils, thereby stimulating autoantibody production by B cells (9, 107).

Mast cells express IgG receptors (FcγRIII, FcγRIIa, and FcγRI) and C3a and C5a receptors (C3aRs and C5aRs, respectively), which are important for complement activation and IgG-induced inflammation (108, 109).

#### 2.2.4 Keratinocytes

Keratinocytes are implicated in the pathogenesis of BP. The separation of keratinocytes, induced by BP autoantibodies *via* Rac1/proteasome activation, is critical for blister formation (110). Keratinocytes secrete thymus and activation-regulated chemokine (TARC/CCL17), a ligand for CCR4 and CCR8 important for the migration of these receptor-expressing cells (111). TARC is increased in BP plasma and lesions (112, 113). Keratinocytes express tissue plasminogen activator (tPA) after BP180 autoantibody activation (114). tPA, a component of the plasminogen/plasmin system, may interact with MMP-9 or NE to promote inflammation (115, 116).

#### 2.2.5 Complement

Linear complement deposition along the dermal–epidermal junction occurs in >80% of BP patients (117). A role for the classical and, to a lesser degree, alternative complement pathways in BP blister formation has been reported (118).

The anti-BP180 NC16A IgG serum level is significantly higher in patients with C3 deposition, and patients without blisters have significantly fewer C3 deposits (117). Antigen-IgG1 autoantibodies binding to the BMZ trigger complement activation (21). C3 activation at the dermal–epidermal junction leads to the formation of chemotactic peptides (activated third component of complement [C3a] and activated fifth component of complement [C5a]) and the recruitment of neutrophils, eosinophils, and macrophages to this site (85, 108, 118, 119). The activated fifth component of complement (C5a), along with C5a receptor 1 (C5aR1), but not C5aR2, plays a role in the early phase of BP by promoting neutrophil standstill and leukotriene release in the endothelium; in turn, this induces neutrophil migration to the interstitial space *via* an autocrine/paracrine circuit (120, 121). *C5*- and *C4*-deficient mice showed no blisters after mCol17 IgG injection (118). Also, no BP lesions appeared in non-C1q-binding anti-hCol17 IgG1 mutation COL17 humanized mice (21). A targeted C1s inhibitor is under evaluation as a BP treatment in a clinical trial (122).

## 3 Interactions among immune cells

### 3.1 Clinical heterogeneity may be associated with different types of pathogenesis

The mechanism of blister formation in BP is unclear. Some BP patients primarily show eczema lesions for several years (123), and others have BP autoantibodies but not lesions (124, 125). Some BP patients present with blisters and bullous without obvious erythema, whereas others show patchy erythema with few or no blisters (126). In most BP patients, autoantibodies can be detected using commercial products, although in a small proportion of patients, the tests are negative (125). Most infiltrating immune cells in BP are eosinophils, along with some neutrophils (127, 128) and other cell types. Some BP patients respond well to topical steroids, whereas others need systemic steroids and immunosuppressants. Also, refractory BP patients respond differently to rituximab, dupilumab, and omalizumab (11, 129). Different immune cells induce inflammation in various BP models (89, 96, 130). Whether autoantibodies or inflammatory cells are more important in the pathogenesis of BP is unclear. The pathogenesis of BP may involve several immune pathways and infiltrating cell types; clinical presentations and the response to different treatment regimens vary (131–133).

### 3.2 Crosstalk among immune cells

**Figure 1:**
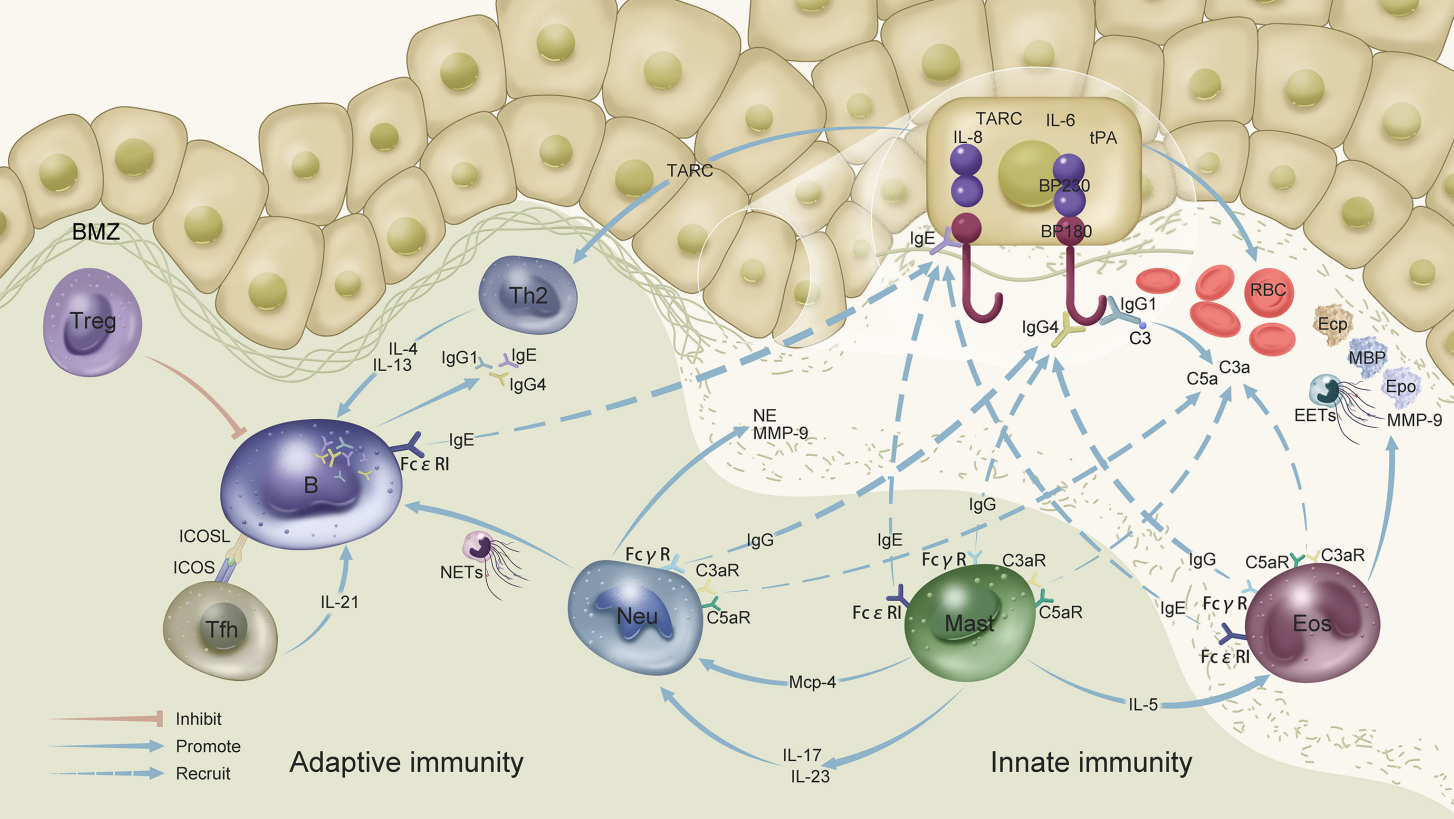
Innate and adaptive immunity in bullous pemphigoid: B cells produce IgE, IgG1, and IgG4 autoantibodies to bind antigens to the BMZ. Antigen–IgG1 binding to the BMZ triggers complement activation. C3 activation at the dermal-epidermal junction leads to the formation of chemotactic peptides (C3a and C5a), which recruit neutrophils and eosinophils and induce mast cell degranulation, thereby contributing to blister formation. Antigen–IgG4 binding leads to the recruitment of neutrophils and eosinophils and, consequently, to the release of proteolytic enzymes. BP180-specific IgG autoantibodies modulate IL-6, IL-8, and tPA expression in human keratinocytes. TARC/CCL17 secreted by keratinocytes can recruit and activate Th2 cells. IgE autoantibodies amplify the inflammation in BP by interacting with eosinophils, mast cells, and B cells. IgE autoantibodies could also induce BP180 internalization in basal keratinocytes, thereby suppressing their adhesion. Tfh cells promote the production of high-affinity autoantibodies from B cells via regulation by IL-21 and ICOS-ICOSL. Activated Th2 cells secrete IL-4, which regulates IgG isotype and IgE switching. Mast cells activated by IgE degranulate and release IL-5 to promote eosinophil accumulation and activation. Eosinophils are attracted to the BMZ by IgG autoantibodies and complement fixation, and degranulate after interacting with IgE. Eosinophils secrete EETs and toxic granule proteins, including ECP, MBP, EPO, and MMP-9, which are involved in the local inflammatory cascade. In addition, activated mast cells release MCP-4, which activates neutrophils. Activated neutrophils release cytokines and proteases, including NE and MMP-9, which degrade the extracellular matrix and split dermal-epidermal junctions. Neutrophils also release NETs and stimulate autoantibody production by B cells. Neutrophils and mast cells release IL-17 and IL-23, thereby significantly enhancing MMP-9 and NE production by neutrophils. tPA, a component of the plasminogen/plasmin system secreted by keratinocytes, may interact with MMP-9 or NE to promote inflammation. What’s more, tPA, MMP-9, NE and eosinophils all could lead to the activation of coagulation system, inducing thrombotic and bleeding risk of skin. BMZ, basement membrane zone; C3, complement 3; EETs, eosinophil extracellular traps; MMP-9, matrix metalloproteinase-9; NE, neutrophil elastase; NETs, neutrophil extracellular traps; MCP-4, mast cell protease-4; C3a, activated third component of complement; C5a, activated fifth component of complement; C3aRs and C5aRs, C3a and C5a receptors, respectively; RBC, red blood cell; EOS, eosinophil; Th, helper T cell; Tfh, T follicular helper cell; Neu, neutrophil; B, B cell; Mast, mast cell; MBP, major basic protein; ECP, eosinophil cationic protein; EPO, eosinophil peroxidase; TARC, thymus and activation-regulated chemokine; tPA, tissue plasminogen activator.

Autoantibody binding to pathogenic antigen cause the separation of the BMZ in a complement-dependent or -independent manner (5, 21, 85, 118) (**Figure 1**). Antigen–IgG1 binding to the BMZ triggers complement activation. C3a and C5a induce neutrophil and eosinophil chemotaxis, as well as mast cell degranulation, which in turn induce inflammation and blister formation (21, 109, 118, 121).

Antigen–IgG4 induced the separation of the BMZ through a complement-independent pathway (5, 22, 134). The antigen–antibody combination leads to the recruitment of neutrophils and eosinophils in BP, and, consequently, to the release of proteolytic enzymes (5). Eosinophils trigger the separation of the BMZ in the presence of IgE or IgG (84, 86, 89). The activation of intracellular pathways leads to pyrolytic hemidesmosomes and attracts immune cells, inducing the inflammatory cascade.

BP180 IgG autoantibodies modulate IL-6, IL-8, and tPA expression in human keratinocytes (114). tPA activates plasmin and MMP-9. Activated MMP-9 hydrolyzes the α1 protease inhibitor, which is an NE inhibitor, thus enhancing NE activity (115, 116). Keratinocytes secrete TARC/CCL17, thereby activating Th2 cells (111).

Tfh cells promote the production of high-affinity autoantibodies by B cells in germinal centers. Activated Th2 cells in BP secrete IL-4, which regulates IgG isotype and IgE switching, thereby amplifying the production of IgG4 and IgE (56, 135, 136). IgE autoantibodies induce BP180 internalization in basal keratinocytes, which reduces their adhesion (30). IgE autoantibodies interact with eosinophils, mast cells, basophils, and B cells *via* CD23 and FcϵRI (39–42). Mast cells, activated by IgE, degranulate and release IL-5 to promote eosinophil accumulation and activation (8, 104). Eosinophils are attracted to the BMZ by IgG autoantibodies and complement fixation (85) and degranulate after interacting with IgE (86).

Eosinophils secrete EETs and toxic granule proteins, such as ECP, MBP, EPO, and MMP-9, after exposure to GM-CSF, IL-5, IFN-γ, eotaxin, and TSLP, which are involved in the local inflammatory cascade (84, 88–91). In addition, activated mast cells release MCP-4, which activates neutrophils. Activated neutrophils release cytokines and proteases, including NE and MMP9, which degrade the extracellular matrix and split dermal–epidermal junctions (81, 98, 99, 106). tPA, MMP-9, NE, and eosinophils can all lead to the activation of the coagulation system, inducing possible thrombotic and bleeding risks of skin and internal organs (137). Neutrophils also release NETs and stimulate autoantibody production by B cells (9, 107). Neutrophils, lymphocytes, monocytes, and mast cells release IL-17 and IL-23, thereby significantly enhancing MMP-9 and NE production by neutrophils (81, 138, 139). Mast cells express IgG receptors (FcγRIII, FcγRIIa, and FcγRI), C3a, and C5aRs, which interact with complement and IgG (108, 109).

In conclusion, a variety of immune cells and cytokines are implicated in the pathogenesis of BP, including T cells, B cells, eosinophils, mast cells, neutrophils, complement, and plasminogen/plasmin. However, the underlying pathways require further investigation.

## Author contributions

TY wrote the manuscript, and ZZ revised the article for important intellectual content. All authors contributed to the article and approved the submitted version.
